# Aortic stenosis and right ventricular dysfunction

**DOI:** 10.1007/s10554-023-02986-9

**Published:** 2023-11-11

**Authors:** Pavol Fulop, Gabriel Valocik, Marianna Barbierik Vachalcova, Pavol Zenuch, Lenka Filipova

**Affiliations:** 1 1st Department of Cardiology, Medical Faculty of University Pavol Jozef Safarik, East Slovak Institute of Cardiovascular Diseases, Ondavska 8, 040 11 Kosice, Slovakia; 2 Department of Internal Medicine, Medical Faculty of University Pavol Jozef Safarik, Hospital Agel Kosice-Saca, Lucna 57, 040 18 Kosice-Saca, Slovakia; 3 2nd Department of Cardiology, Medical Faculty of University Pavol Jozef Safarik, East Slovak Institute of Cardiovascular Diseases, Kosice, Slovakia

**Keywords:** Aortic stenosis, Low gradient severe aortic stenosis, High gradient severe aortic stenosis, Transesophageal 3D echocardiography, Right ventricular dysfunction

## Abstract

At the present time, right ventricular function in patients with aortic stenosis is insufficiently taken into account in the decision-making process of aortic valve replacement. The aim of our study was to evaluate significance of right ventricular dysfunction in patients with severe aortic stenosis by modern 3D echocardiographic methods. This is prospective analysis of 68 patients with severe high and low-gradient aortic stenosis. We evaluated function of left and right ventricle on the basis of 3D reconstruction. Enddiastolic, endsystolic volumes, ejection fraction and stroke volumes of both chambers were assessed. There were more patients with right ventricular dysfunction in low-gradient group (RVEF < 45%) than in the high-gradient group (63.6% vs 39%, *p* = 0.02). Low-gradient patients had worse right ventricular function than high-gradient patients (RVEF 36% vs 46%, *p* = 0.02). There wasn’t any significant correlation between the right ventricular dysfunction and pulmonary hypertension (r = − 0.25, *p* = 0.036). There was significant correlation between left and right ejection fraction (r = 0.78, *p* < 0.0001). Multiple regression analysis revealed that the only predictor of right ventricular function is the left ventricular function. According to our results we can state that right ventricular dysfunction is more common in patients with low-gradient than in high-gradient aortic stenosis and the only predictor of right ventricular dysfunction is left ventricular dysfunction, probably based on ventriculo-ventricular interaction. Pulmonary hypertension in patients with severe AS does not predict right ventricular dysfunction.

## Introduction

According to the EURObservational Research Programme Valvular Heart Disease II Survey, aortic stenosis (AS) is the most common valvular defect in Europe. Based on the monitoring of 5219 patients in this programme, 2152 patients (41.2%) suffered from severe AS [1]. AS is common mainly in the elderly after the age of 75 with a prevalence of 12%. Severe AS occurs with a prevalence of 1–3% in people over 65 years of age, with an increasing prevalence with age [2, 3]. Despite advances in knowledge of clinical, genetic and molecular mechanisms of the disease, surgical or interventional aortic valve replacement is the only method of treatment [4]. Currently, the severity of AS is assessed by several criteria. The most common is a classification by haemodynamic parameters [(peak velocity, mean gradient, aortic valve area (AVA)]. The patients with severe low-gradient AS belong to a special group. These patients have a low mean gradient (< 40 mmHg) and aortic valve area, which corresponds to severe AS (AVA < 1 cm^2^). Current recommendations, which define algorithms in the decision-making process regarding aortic valve replacement, don´t take into account the anatomical and functional consequences of AS on other parts of the heart, excluding systolic function of the left ventricle (LV) [5]. Recently, a new anatomical and functional classification of the patients with severe AS has been proposed [6]. In addition to the LV, it takes into account the function of the right ventricle (RV). For decade, the role of the RV has been downplayed by the attribute of a forgotten chamber. Several research studies have confirmed that RV dysfunction in patients with severe AS is a significant predictor of mortality, and RV function should be systematically assessed [7–9]. The RV function in the aforementioned research studies was assessed by various parameters of two-dimensional and one-dimensional echocardiography, which are burdened with errors and inaccuracies when assessing the RV function. To date, there aren´t any studies assessing the RV function in patients with severe AS by means of modern 3D echocardiographic methods, which are characterized by higher accuracy compared to 2D echocardiographic methods. Therefore, the aim of this study was to evaluate the role of RV dysfunction in patients with severe AS by modern 3D echocardiographic methods.

## Methods: patients

**Fig. 1:**
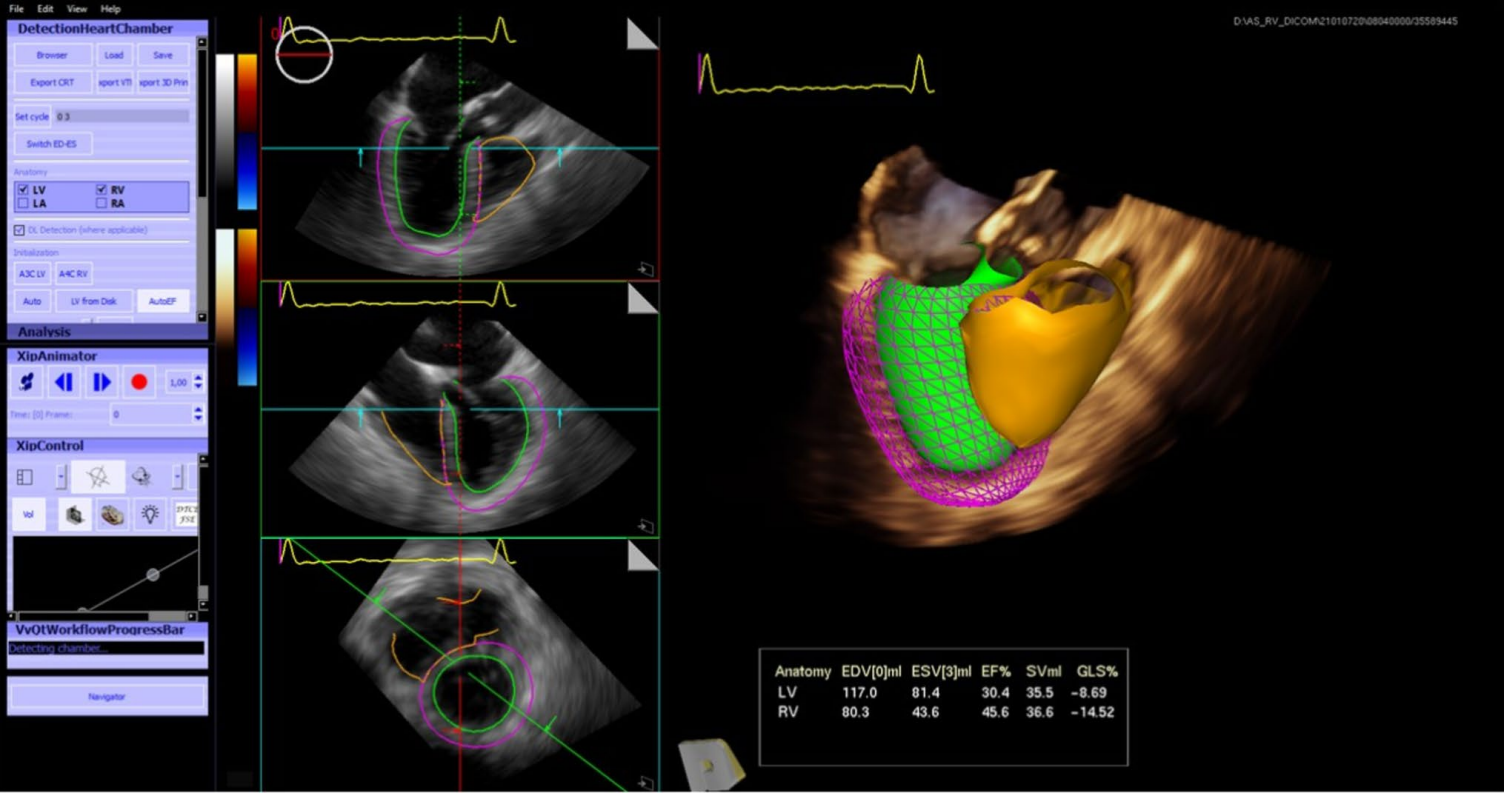
3D analysis of the function of the left ventricle (LV), right ventricle (PC), end-diastolic volumes (EDV), end-systolic volumes (ESV), stroke volumes (SV), ejection fractions (EF)

This is prospective analysis of patients with severe AS. Patients enrolled to the study suffered severe AS and had been performed transoesophageal 3D echocardiography (3D TEE) using high quality 3D imaging of the LV and RV (the ultrasound system Siemens Acuson SC 2000 Prime, Mountain View, CA, USA). The patients were examined at the Department of Echocardiography, 1st Department of Cardiology of the East-Slovak Institute for Cardiovascular Diseases and at the Faculty of Medicine, P. J. Safarik University in Košice. Inclusion criteria included severe AS, high-gradient (HG), and low-gradient (LG). High-gradient AS was defined haemodynamically as AS with maximum transvalvular velocity of > 4 m/s, and/or mean gradient of > 40 mmHg and AVA of < 1 cm^2^. Low-gradient AS was defined haemodynamically as AS with maximum transvalvular velocity of < 4 m/s, mean gradient of < 40 mmHg and AVA of < 1 cm^2^. Exclusion criteria included pseudostenosis, which was evaluated according to the applicable recommendations, in particular by dobutamine stress echocardiography and also by evaluation of the aortic valve calcium score using CT examination [10]. Besides the primary echocardiographic parameters, which are commonly used in AS evaluation, the LV and RV function on a basis of 3D reconstruction using a prototype program (Siemens, EchoBuildR v3.5.0., Mountain View, CA, USA) for a 3D analysis of the cardiac cavities were assessed. It is an automatic 3D analysis of the LV and RV function where the end-diastolic and end-systolic volumes of the LV and RV, EF and stroke volumes of both ventricles were assessed (Fig. 1). Contours of right ventricule were marked for the 4 chamber, sagital and coronal views in the systole and diastole. Contour drawing used the point-to-point method. After evaluation and confirmation of countours 3D model of RV was obtained. Left ventricule was assessed using a single heartbeat. In left ventricular analysis knowledge-based workflow was used to automatically extract 3 views (apical 4, 3 and 2 chamber) and the transverse view. Pulmonary hypertension (PH) was assessed echocardiographically from tricuspid regurgitation according to applicable recommendations [11].

Prior examination, each patient signed an informed consent form. All procedures followed were in accordance with the ethical standards of the responsible committee on human experimentation (institutional and national) and with the Helsinki Declaration of 1975, as revised in 2008.

### Statistical methods

Qualitative data were assessed as mean ± standard deviation. Quantitative data were presented in percentage. Data were compared by ANOVA test or by × 2 test. The relationship between particular parameter was assessed by linear or multiple regression analysis. Analyses with value of *p* < 0.05 were considered as statistically significant.

## Results

Primary echocardiographic data are presented in the Table 1.

**Table 1:** Means ± SD

	ALL (n = 67)	HG (n = 45)	LG (n = 22)	*p*
Age	73.19 ± 9.6	72.09 ± 10.42	75.45 ± 7.45	0.211
AVA VTI	0.67 ± 0.28	0.61 ± 0.27	0.78 ± 0.26	0.001
AVA/BSA	0.35 ± 0.15	0.32 ± 0.14	0.40 ± 0.15	0.004
AoVmax	4.14 ± 0.81	4.55 ± 0.53	3.29 ± 0.61	< 0.001
PG	71.10 ± 26.61	83.94 ± 21	44.83 ± 14.78	< 0.001
MG	43.48 ± 16.64	51.28 ± 13.85	27.51 ± 8.31	< 0.001
SV LVOT	63.91 ± 27.36	65.98 ± 30.34	59.47 ± 18.04	< 0.001
SVi LVOT	32.94 ± 14.56	34.84 ± 15.60	29.06 ± 10.75	< 0.001
Flow	168.29 ± 82.56	179.84 ± 91.41	145.18 ± 56.68	0.06
LV EF	42.08 ± 15.93	44.84 ± 15.24	36.43 ± 16.56	0.011
EDV LV	137.53 ± 54.81	137.26 ± 60.99	138.08 ± 40.64	0.41
ESV LV	83.82 ± 48.54	80.55 ± 53.00	90.51 ± 38.78	0.17
SV LV	54.11 ± 23.55	56.77 ± 24.00	48.65 ± 22.28	0.078
RV EF	42.71 ± 17.08	45.99 ± 15.90	36.01 ± 18.05	0.02
EDV RV	100,37 ± 38.96	99.84 ± 45.35	101.44 ± 20.68	0.3
ESV RV	60.42 ± 37.70	57.58 ± 41.98	66.25 ± 26.28	0.16
sPAP	31.42 ± 15.92	31.60 ± 16.82	31.05 ± 14.47	0.36

*AVA VTI* aortic orifice area according to VTI, *PG* peak gradient, *MG* mean gradient, *SV* stroke volume, *LVOT* left ventricular outflow tract, *EF* ejection fraction, *LV* left ventricle, *RV* right ventricle, *EDV* end-diastolic volume, *ESV* end-systolic volume, *GLS* global longitudinal strain, *sPAP* systolic pressure in the pulmonary artery

**Fig. 2:**
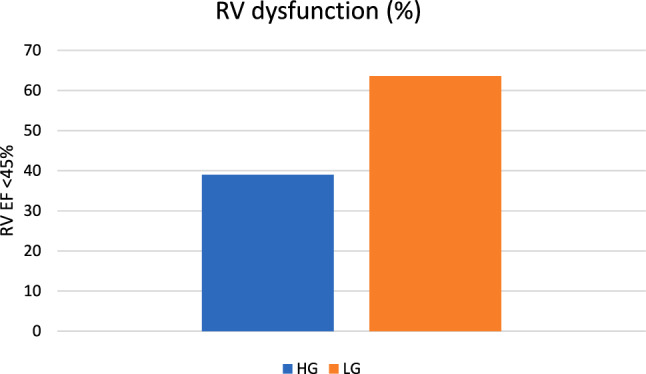
Percentage representation of patients with right ventricular (RV) dysfunction, defined as RV EF < 45%, in the group with low-gradient aortic stenosis (LG) and high-gradient aortic stenosis (HG)

**Fig. 3:**
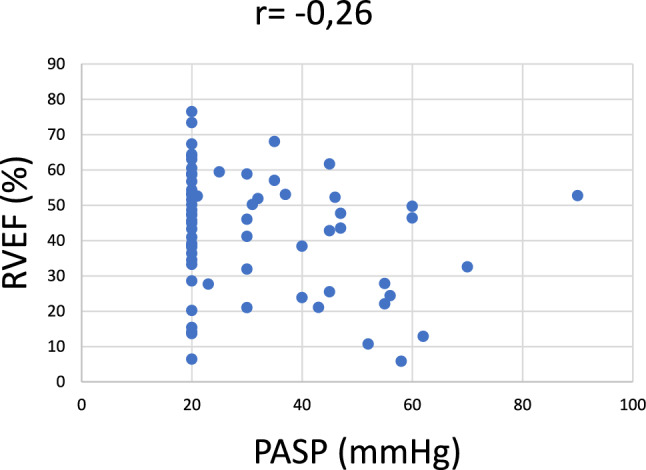
Correlation between right ventricule ejection fraction (RVEF) and pulmonary artery systolic pressure (PASP)

**Fig. 4:**
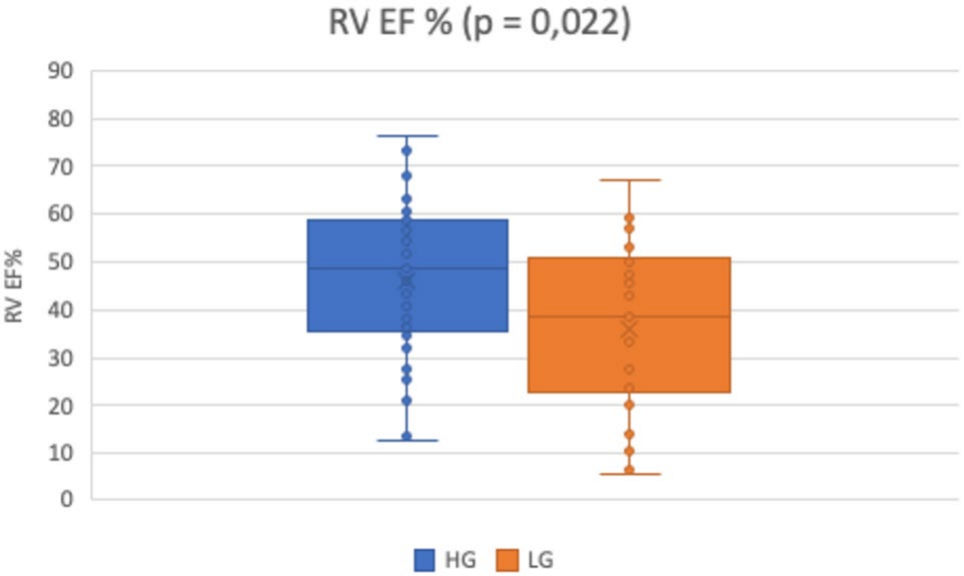
Right ventricular ejection fraction (RV EF) in patients with high-gradient aortic stenosis (HG) and low-gradient aortic stenosis (LG)

**Fig. 5:**
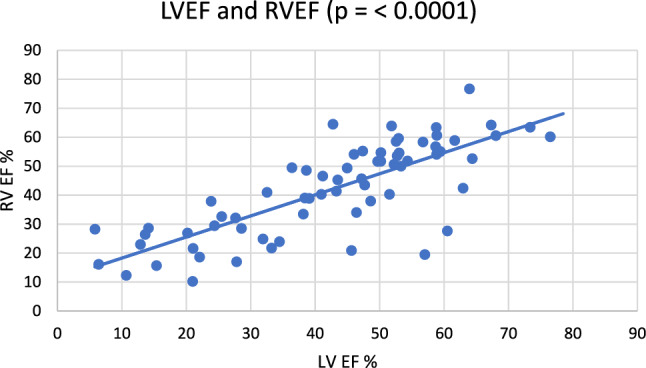
Correlation graph of right ventricular (RV EF) and left ventricular (LV EF) function

In the patient cohort, there were significantly more patients with the RV dysfunction (characterized by EF RV < 45%) in the LG AS group (63.6%) than in the HG AS group (39%) (*p* = 0.02, Fig. 2). There wasn´t any significant correlation between the RV dysfunction and PH (r = − 0.25, *p* = 0.036, Fig. 3). But the patients with LG AS had worse RV function (36% ± 18) that the patients with HG AS (46% ± 15.9) (*p* = 0.02, Fig. 4). Multiple regression analysis showed that the LV function is the only predictor of the RV function (*p* = 0.02, Table 2). Significant correlation was found between LV EF and RV EF (r = 0.78, *p* < 0.0001, Fig. 5).

**Table 2:** Multiple regression analysis model, where right ventricule function is the dependent variable

	β	*p*-value	Lower 95%	Upper 95%
Intercept	15.34	0.49	− 29.16	59.84
LV EF (%)	0.57	0.02	0.08	1.05
AoV Mean Gradient (mmHg)	0.06	0.83	− 0.52	0.65
AoV Vmax (m/s)	− 1.86	0.71	− 11.98	8.27
AoV Area VTI (cm^2^)	1.03	0.95	− 29.60	31.66
Flow rate (ml/beat)	0.09	0.24	− 0.06	0.24
stroke volume index (ml/m^2^)	0.24	0.47	− 0.42	0.90
EDV LV (ml)	− 0.49	0.34	− 1.50	0.52
ESV LV (ml)	0.42	0.39	− 0.56	1.40
SV LV (ml)	0.17	0.78	− 1.01	1.35
AoV VTI (m)	9.26	0.69	− 37.07	55.60
LVOT Diam (cm)	0.07	0.91	− 1.15	1.29
LVOT CO (l/min)	0.28	0.88	− 3.31	3.87
sPAP (mmHg)	− 0.10	0.33	− 0.29	0.10

*LV EF* left ventricule ejection fraction, *AoV* aortic valve, *Vmax* maximal velocity, *VTI* velocity time integral, *EDV* end-diastolic volume, *ESV* end-systolic volume, *SV* stroke volume, *LVOT* left ventricule outflow tract, *Diam* diameter, *CO* cardiac output, *sPAP* systolic pulmonary artery pressure

## Discussion

This study points to the role of RV function assessment using 3D TEE. It is based on the following facts. Nearly half of the patients (47%) with severe AS were diagnosed dysfunctional RV when assessed as RV EF < 45%. Majority of patients with dysfunctional RV was included in the LG AS subgroup (63.6% vs 39%). Similarly, the patients with LG AS had worse RV function than the patients with HG AS (36% ± 18 vs 46% ± 15.9). The LV function (i.e. LV EF) was the only predictor of the RV function. Pulmonary hypertension didn’t show significant impact on the RV function. There was significant correlation between the LV EF and RV EF.

3D echocardiography is currently considered to be an accurate method in the assessment of function and volumes, especially in the case of the LV. Fully automatic assessment modalities of volumes and EF LV were compared with values of volume and EF from magnetic imaging which is considered to be a golden standard [12]. Both, end diastolic and end systolic volumes were moderately undervalued when assessed by 3D echocardiography, but the values of EF were almost identical. Although, LV function assessment by 2D echocardiography is recommended, the clinics, which have experience of using 3D echocardiography, are advised to assess the volumes and LV function by 3D echocardiography. In the case of the RV, according to current recommendations, the RV function is assessed integratively, which implies the use of multiple parameters, such as Doppler, one-dimensional and two-dimensional echocardiography, in the RV function assessment [13]. This results from the inaccuracy of these methodologies. Due to the irregular shape of the RV, the measurement of the volumes and EF RV is more complex than in the case of the LV, which has a simpler geometrical shape. When comparing the 3D echocardiography with MR in volumetry or EF RV assessment, the volumes were significantly undervalued by 3D cardiography, but no significant deviation was recorded when measuring EF by means of 3D echocardiography [14]. According to our knowledge, this is the first study in which LV and RV functions were assessed in relation to AS using 3D echocardiography.

Close relationship between RV and LV function is most likely conditioned by ventricular-ventricular interaction (VVI). The term VVI describes cumulative effect of filling, function, geometry, and synchrony of one ventricle on filling, function, geometry and synchrony of the contralateral ventricle [15]. On usual conditions, a significant part of RV mechanical work is generated by contraction of the LV. VVI plays a significant role mainly in ventricular pressure and volume overload which influences the course and prognosis of the disease. The RV and LV have common interventricular septum (IVS), pericardium and circular muscle fibres which surround both ventricles. The RV free wall is mainly composed of the transverse fibres, which ensure the transversal compression of the RV. It contributes in 20–30% to the RV function. The septum, which belongs to both the RV and LV, is thus biventricular in its nature. It composes approximately 40% of the pure weight of the heart (LV = 38 ± 5%, IVS = 35 ± 4% and RV = 27 ± 1%). The IVS is composed of striated descending and ascending muscle fibres, which form an angle of 60°. Such IVS arrangement/architecture is highly effective because it is involved in the RV function of 70–80%. In patients with pulmonary hypertension but functional IVS, RV function doesn´t decrease significantly, because strong IVS compensates for a dysfunctional RV free wall, which is only partially involved in overall RV function. On the contrary, in patients with both PH and impaired IVS, more significant RV failure occurs, because a larger part of the RV is dysfunctional, i.e. IVS, and thus, the RV free wall can´t compensate IVS dysfunction. Another presumed mechanism of RV and LV interaction is that with significant RV or LV dilatation, the angle between striated muscle fibres decreases (< 60°), and thus effectivity of contraction decreases [16].

The role of the RV function assessment in patients with AS was demonstrated by Eleid et al. [17]. In the cohort of 44 patients who underwent transcatheter aortic valve replacement (TAVR), they found out that immediately after aortic valve replacement and thus relieve of stenosis, the RV function improved in terms of effusion in the RV and systolic velocity of the tricuspid ring. After TAVR, there was no decrease of pressure in the pulmonary trunk, so modification of the RV function wasn´t caused by modification of pulmonary hypertension. The authors assumed that the improvement of RV function was due to VVI. In general, there is a tendency to avoid the intervention when there is a combination of AS and RV dysfunction, although the modification of RV function after TAVR presented in this study suggests the opposite, i.e. the need of intervention; TAVR, which also results to modification of RV function. There are several similar data between our study and the aforementioned study. For example, our cohort consisted of 47% patients with RV dysfunction compared to the study of the aforementioned authors which included 50% of patients with RV dysfunction. Similarly to our findings, the authors didn’t confirm the significant relationship between pulmonary hypertension and RV dysfunction. They claim that the most likely mechanism, which causes RV dysfunction, is LV function during VVI.

Using a solid finite element model of the heart with AS, Wisneski et al. in their study described the highest systolic strain in the area of the basal IVS segment [18]. It can be assumed that in patients with severe AS, the IVS is the most burdened area during pressure overload. This may be the reason for one of the mechanisms of the RV dysfunction.

Forsberg et al. observed the patients with severe AS who underwent TAVR and surgical aortic valve replacement (SAVR) [19]. They assessed the echocardiographic parameters such as atrioventricular plane displacement (AVPD) at the level of the LV lateral wall, IVS, and RV free wall. At these levels, they also monitored the systolic velocities (PVS, peak systolic velocity) assessed by tissue Doppler. These two cohorts of patients were matched by age, sex, and EF LV. AVPD and PSV after TAVR, or SAVR in the area of the LV lateral wall increased in both groups. In the IVS area, AVPD nor PVS increased in the subgroup of patients after SAVR. The RV free wall, AVPD and PSV didn´t change in patients after TAVR but decreased in the cohort of patients after SAVR. The authors conclude that the RV impairment doesn´t occur after TAVR but SAVR. The RV is more protected by TAVR than SAVR. According to some data, the RV dysfunction may persist even 1 year after the cardiosurgical procedure [20]. There are several presumable mechanisms of the RV impairment after the cardiosurgical procedures. Firstly, the drains in front of the RV, post-surgical adhesions, the right ventricle impairment (wall oedema, mechanical trauma) during cannulation for extracorporeal circulation. The LV is more protected by hypothermia than the RV, which is more exposed to the ambient temperature of the surgical theatre, while the procedure is being performed [21]. Secondly, the insufficient perfusion of the RV myocardium during retrograde cardioplegia. Allen et al. assessed perfusion of the RV myocardium using contrastive echocardiography. The study results confirmed that the LV and IVS were threefold to fourfold better perfundated than the RV free wall [22].

## Limitations

The main limitation of our study is that pulmonary hypertension wasn´t measured by invasive methods but indirectly from the echocardiographic assumption of pressures in the pulmonary trunk using the tricuspid regurgitation during Doppler examination.

## Conclusion

Based on the findings, we can conclude that right ventricle dysfunction is more common in patients with LG AS than in the patients with HG AS. The LV dysfunction is the only predictor of RV dysfunction; most probably it is based on VVI. Pulmonary hypertension in patients with severe AS doesn’t predict worse RV function. Potential clinical application of presented findings is a preference of TAVR over SAVR in the patients with RV dysfunction.

## Data Availability

The datasets used and/or analysed during the current study are available from the corresponding author on request.
